# Beyond clinical care: direct, indirect, and non-physician activities and their implications for workforce planning and postgraduate medical education in a university neurosurgical department

**DOI:** 10.1007/s00701-026-07084-y

**Published:** 2026-11-13

**Authors:** Dorothee Spille, Elisa Regenhardt, Ann-Katrin Bruns, Stefan Geißler, Markus Wenning, Alexander Friedrich, Walter Stummer

**Affiliations:** 1 Department of Neurosurgery, University Hospital Münster, Münster, Germany; 2 University Hospital Münster (UKM), Münster, Germany; 3 Medical Association Westfalen-Lippe, Münster, Germany; 4 Department of Neurosurgery, University Hospital Hamburg-Eppendorf, Martinistraße 52, 20251 Hamburg, Germany

**Keywords:** Physician staffing, Reimbursement in healthcare, Quality of care, Location-specific process optimization, Recording of working hours

## Abstract

**Background:**

Realistic determination of physician staffing requirements is essential to ensure high-quality patient care and the long-term sustainability of healthcare systems under increasing financial constraints. However, empirical data on medical workforce planning remain scarce. Using a neurosurgical university department as an example, we sought to quantify physician staffing requirements and compare different approaches to workforce calculation.

**Methods:**

Physician staffing requirements were assessed using three complementary approaches: (1) an economic–normative approach based on DRG reimbursement and the InEK (Institute for the Hospital Renumeration System/ Institut für das Entgeltsystem im Krankenhaus) cost matrix; (2) a deductive–institutional approach incorporating expected clinical workload as well as vacation and sick leave; and (3) an empirical time-motion analysis based on real-time recording of physicians’ activities. In addition to direct patient care, indirect medical tasks and non-physician duties were systematically documented.

**Results:**

The DRG-based calculation yielded a reimbursable staffing requirement of 15.5–19.5 full-time equivalents (FTEs). After accounting for indirect clinical activities, vacation, and sick leave, the deductive–institutional approach estimated a requirement of 23.5 FTEs. However, the empirical analysis demonstrated that a substantial proportion of physicians’ working time was devoted to activities not directly related to patient care, particularly on-call duties (24.7%) and ward-related organizational tasks (15.6%). These activities were insufficiently captured by the economic and deductive models and would require additional staffing resources.

**Conclusion:**

Physician staffing requirements are inadequately reflected by reimbursement-based DRG calculations. Realistic workforce planning requires consideration of indirect clinical activities, organizational duties, and institution-specific workflow analyses. Although these estimates were derived within the German healthcare system, neurosurgical departments internationally are likely confronted with similar demands arising from non-clinical and administrative responsibilities. Identifying and reducing such tasks may represent an important opportunity to improve efficiency and optimize physician workforce utilization.

**Supplementary Information:**

The online version contains supplementary material available at https://doi.org/10.1007/s00701-026-07084-y.

## Introduction

How many physicians are required to ensure adequate hospital care and to provide appropriate training for resident physicians? At least the question of physician workload is an actively debated issue in Germany. A newly negotiated collective agreement stipulates a reduction in the weekly working hours of physicians at university hospitals from 42 to 40 h. At the same time, the hospital reform legislation calls for systematic physician staffing benchmarks based on the Hospital Care Improvement Act (*Krankenhausversorgungsverbesserungsgesetz*, KHVVG), including the development of a nationally standardized instrument for assessing physician staffing requirements [8, 18, 30]. The methodological foundation for this is the Physician Staffing Assessment System developed by the German Medical Association (*Ärztliches Personalbemessungssystem der Bundesärztekammer*, ÄPS-BÄK), which systematically captures physician activities and calculates the actual staffing needs of a hospital. This instrument is currently being piloted nationwide to evaluate its practical applicability, comparability, and impact on quality of care and working conditions. The results are intended to be translated into legally binding regulations, establishing transparent, specialty-specific, and verifiable standards for physician staffing [13]. The ÄPS-BÄK represents a deductive approach in which the required resource allocation is derived from a theoretical reconstruction of work processes and an anticipated workload. In practical application, the ÄPS-BÄK relies predominantly on existing routine data, such as case volumes, service profiles, duty roster models, and structural characteristics of clinical departments. In addition, organizational information is collected on a one-time or periodic basis from medical directors or controlling units. On this data basis, the system automatically calculates the required physician time and staffing levels, differentiated by qualification level and area of work. This is consistent with the approach underlying the WHO's Workload Indicators of Staffing Need (WISN) instrument, which determines staffing requirements through systematic activity-based data collection among healthcare workers [29]. Given the high complexity and the heterogeneity of organizational and infrastructural conditions across hospitals worldwide, it appears reasonable to additionally incorporate site-specific care data into staffing assessments, within the framework of a deductive-institutional approach [2, 21].

In everyday clinical practice, numerous confounding factors must be taken into account that cannot be fully captured as discrete physician activities — for example, travel time between locations, the coordination of diagnostic procedures and interventions, logistical tasks related to patient accommodation and transport, and operational workflow planning. These components are currently only incompletely reflected in deductive calculation approaches. Nevertheless, they substantially shape the day-to-day clinical reality for physicians, particularly for residents in postgraduate training, generating high levels of physical and psychological burden and leaving limited time capacity to ensure adequate medical education [26, 35]. Studies consistently show that residents spend only a fraction of their working time on structured educational activities. Most of their shift is absorbed by administrative tasks, documentation, and logistical duties that bear little direct relevance to clinical skill development [3, 22]. As a result, the actual time available for supervised procedures, case discussions, or feedback sessions falls significantly short of what postgraduate training curricula formally require. Various approaches to learning in the clinical setting exist, including mentoring, peer learning, self-directed study, and on-the-job training. Due to the time constraints inherent to clinical work, the predominant form of learning is on-the-job training; at the same time, mentoring represents a critically important component of medical education, serving to ensure high standards of care quality and to minimize clinical errors [1, 7, 32].

It can therefore be assumed that such confounding factors, which vary from hospital to hospital, play a significant role in determining the total workload required and can only be captured empirically, that is, through direct measurement.

The aim of our analysis was to compare a normative-economic approach and a deductive-institutional approach to physician staffing assessment with an empirical measurement approach, in order to evaluate the extent to which actually occurring workload is adequately represented. Furthermore, the study seeks to characterize the workload of physicians in postgraduate training, particularly in the context of on-call and shift duties, and to examine how this workload shapes the quality of medical education.

## Methods

**Fig. 1:**
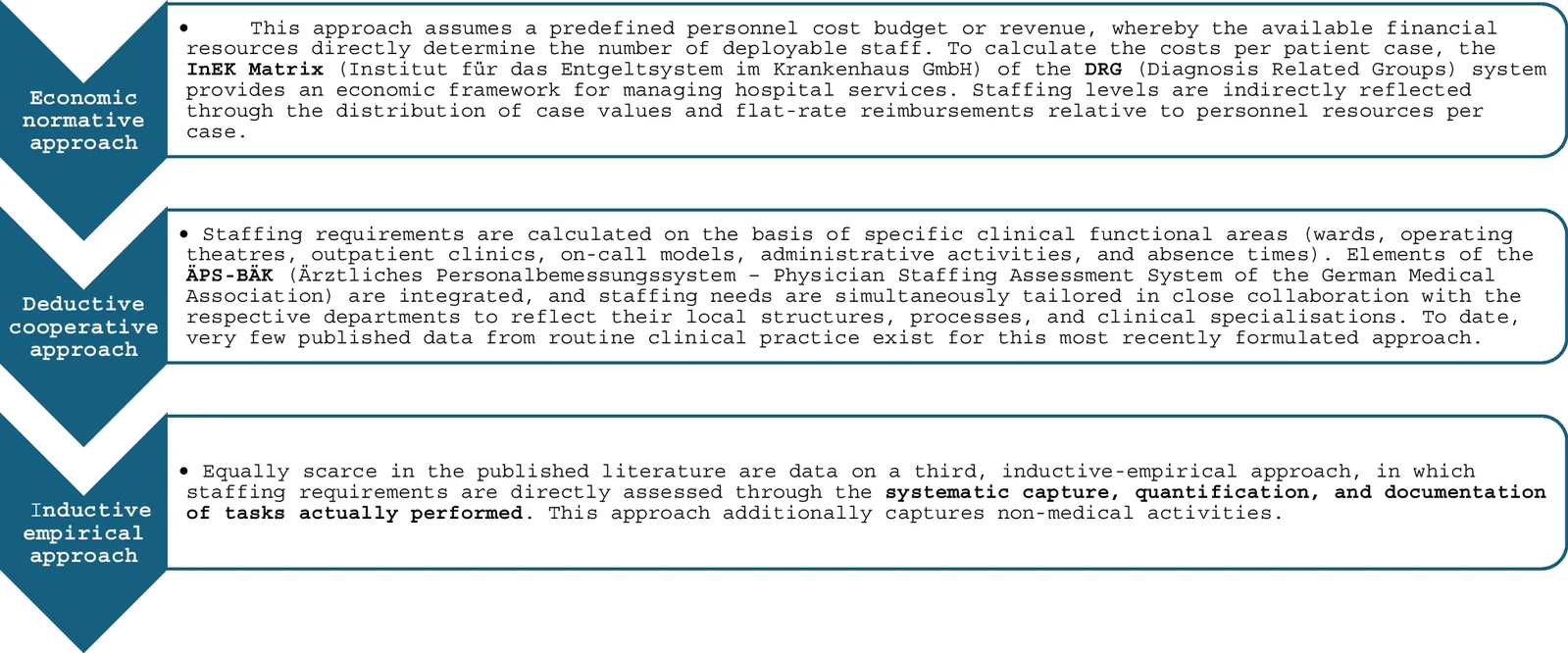
Methodological approaches to workforce requirements assessment

The three methodological approaches to staffing assessment applied here are presented in Fig. 1. The underlying data are derived from structural and process-related information of a medium-sized neurosurgical university hospital in Germany.

A) Economic normative approach based on the InEK cost matrix:

InEK (Institut für das Entgeltsystem im Krankenhaus, i.e., the Institute for the Hospital Remuneration System) is the German institution responsible for calculating and annually updating the case-based DRG (Diagnosis-Related Group) reimbursement rates that determine hospital funding across the German healthcare system.To derive the specific cost and staffing requirements, the InEK-consistent target budget for the medical service was calculated by linking the DRGs relevant to the department with their revenues and the respective InEK cost shares attributed to the medical service. The sum of physician cost allocations yielded the annual total physician budget, which was subsequently divided by the institution's average full costs per full-time equivalent (FTE) in the medical service. The average costs per FTE across all clusters were calculated based on the annual average number of FTEs employed in the medical service during 2024 and the total personnel costs. The revenue shares attributable to the medical service from DRG reimbursements were then determined and divided by the average costs per FTE. In addition to DRG revenues, the revenue shares from all other income streams attributable to the medical service were identified and likewise divided by the average personnel costs. Both FTE values were subsequently summed.

B) Deductive cooperative approach:

All obligatorily staffed functional areas of the department were first systematically identified, followed by the incorporation of site-specific factors, additional task profiles, particular organisational requirements, and previously unaccounted workloads. A central component was operating theatre capacity, whereby the available daily operating time on working days was multiplied by a simultaneity factor of a maximum of 2. The result was then divided by the average daily working hours per physician. For inpatient care, a fixed ratio of assistant physician FTEs per number of beds was applied. For surgical departments, a factor of 2.5 FTEs per 20 beds per ward was used. Outpatient clinic requirements were calculated using the workstation method, based on the opening hours and patient contact profiles of the outpatient facilities as defined by the ambulatory care map. An additional component of this blueprint method was the application of an absence rate averaging 15% for paid leave (annual leave, sick leave, etc.), as well as 2% for internal continuing medical education and other absences. In accordance with German occupational health and safety regulations, physicians who have completed an on-call shift are not available for duty the following day; this was accounted for by applying an additional staffing supplement. Coverage of on-call duties was calculated assuming an average of four on-call shifts per physician per month in accordance with the applicable collective bargaining agreement, with a flat rate of 8.0 FTEs allocated for regular on-call duty and 4.0 FTEs for each remote on-call service, in order to ensure continuous availability and short-notice accessibility of medical personnel.

C) Inductive empirical approach

From March to November 2025, a prospective analysis of medical and non-medical tasks performed by resident physicians at the Department of Neurosurgery, University Hospital Münster, was conducted. Data collection was carried out by an independent doctoral candidate in medicine who accompanied the physicians during their shifts. Time expenditure was recorded using a stopwatch. The classification of activities as "medical" or "non-medical" was performed in accordance with the specialty training regulations of the local Medical Association (Westphalia-Lippe) dated 21 September 2019 (version of 17 June 2023, entered into force on 1 May 2024; Table 1). The analysis was conducted across four settings: (a) 24-hour on-call duty, (b) regular working hours on the general ward, (c) the outpatient clinic, and (d) the operating theatre. In addition, residents completed a survey on workload and perceived quality of training following each completed 24-hour shift.

**Table 1:** Exemplary overview of direct and indirect physician tasks and non-physician tasks

Category	Tasks
Direct Medical Tasks	• History taking and physical examination
	• Diagnosis and differential diagnosis
	• Interpretation of laboratory results, imaging, and functional diagnostics
	• Therapeutic decision-making and indication-setting
	• Surgical procedures and interventions
	• Informed consent consultations
	• Discharge letters, operative reports, and epicrises
	• Consultations and case conferences
	• Ward rounds involving diagnostic and therapeutic decisions
	• Emergency treatment and resuscitation
	• Supervision of residents and trainee physicians
	• Medical delegation decisions
	• Participation in tumour boards
Indirect Medical Tasks	• Coordination of medical workflows
	• Discussion of findings with patients' relatives (medically relevant)
	• Treatment and follow-up planning
	• Interdisciplinary coordination
	• Supervision of delegated tasks
	• Participation in quality circles (medical component)
	• Involvement in certification processes and audits (medical component)
	• Pre- and post-operative planning (physician component)
Non-Medical Tasks Performed by Physicians	• Transit and travel time within the facility
	• Operating room organisation (scheduling and workflow planning)
	• Bed management and patient transport coordination
	• Ordering and scheduling of diagnostic investigations
	• Administrative components of admission and discharge procedures
	• Completion of forms without medical assessment
	• Telephone calls without medical content
	• Equipment and material management
	• Administrative documentation
	• Logistical meetings

### Statistical analysis

Quantitative data were summarised using descriptive statistics, including absolute and relative frequencies for categorical variables, and mean with standard deviation as well as median with interquartile range for continuous variables. Descriptive statistics and figures were generated using IBM SPSS Statistics (Version 31.0; IBM Corp., Armonk, NY, USA) and Microsoft Excel for Microsoft 365 (Microsoft Corporation, Redmond, WA, USA).

## Results

### Normative economic approach

For the on-call duty model a staffing requirement of 12.0 FTEs was calculated, of which 4.0 FTEs were allocated to the senior physician cluster for a senior physician call-out service and 8.0 FTEs to the on-call service (residents and specialist physicians). In parallel, the FTE requirement for all other components representing routine daily operations amounted to 16.9 FTEs, with 10.8 FTEs attributed to the resident and specialist physician cluster and 6.1 FTEs to the senior physician cluster. Comparison of the two FTE requirements revealed that the demand arising from routine daily operations exceeded that of the on-call duty model, both in aggregate and within individual clusters.

### Deductive cooperative approach

By consolidating all components and incorporating workload estimates provided by departmental physicians, a total staffing requirement of 23.5 FTEs was calculated, comprising 12.8 FTEs for resident physicians and 10.7 FTEs for senior and specialist physicians. Again, the staffing requirement for routine daily operations exceeded that of the on-call duty model. Deviations from the FTE value for routine daily operations derived using the blueprint method included 2.8 FTEs for activities such as participation in interdisciplinary centers, attendance at internal conferences (e.g., tumor board meetings), and the performance of consultations, as well as 1.0 FTE for organizational tasks. As the absence rate was calculated as the product of the percentage rate and the preceding values, it increased proportionally.

### Inductive empirical approach

- **On-call shift**

**Fig. 2:**
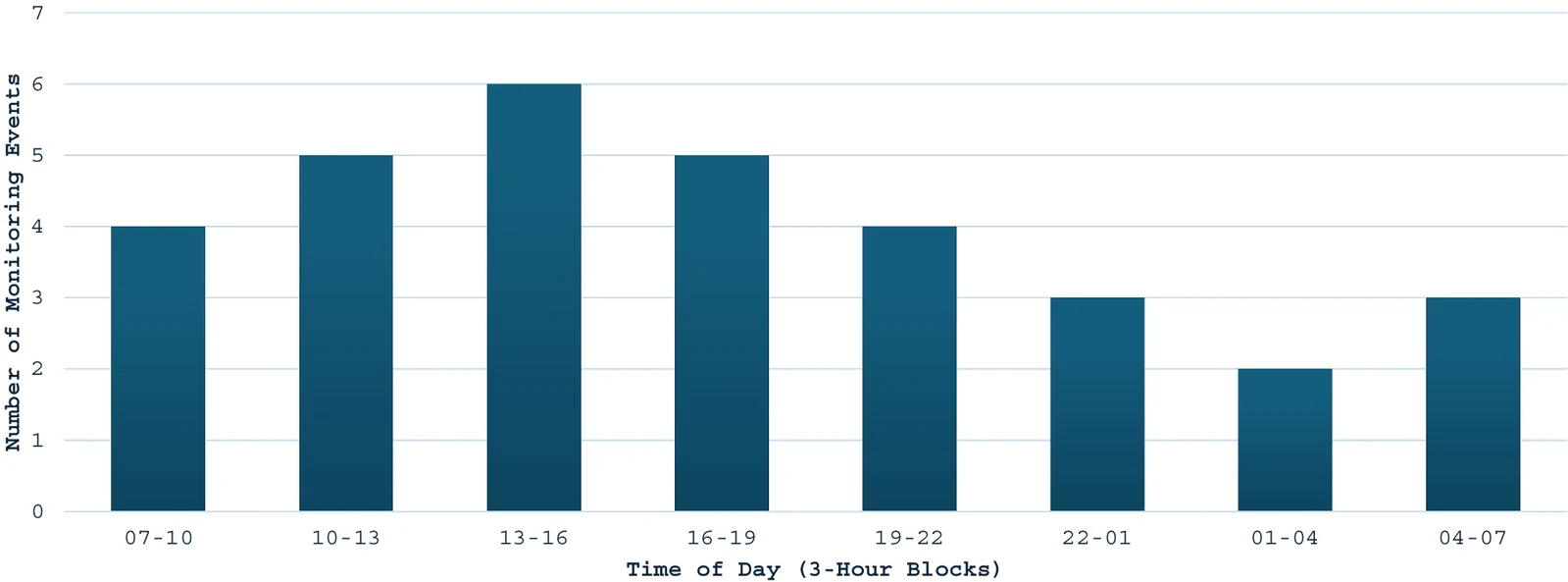
Frequency of monitoring per 3-hour interval

The resident physician group comprised five female and four male participants. The median age was 32 years (IQR: 27–33 years), and the median duration of completed residency training was 3.5 years (IQR: 1.5–6.0 years). A total of 5820 min (97 h) were documented across 24-h on-call shifts. The temporal distribution of observation time across the 24-h period (Fig. 2) demonstrates that individual time intervals were captured with varying frequency. Nonetheless, representative coverage of all time phases was achieved. The majority of observations occurred between 10:00–13:00, 13:00–16:00, and 16:00–19:00, corresponding to typical core working hours. Off-peak periods in the early morning (07:00–10:00) as well as late evening and overnight hours (19:00–22:00, 22:00–01:00, 01:00–04:00, 04:00–07:00) were also covered.

**Fig. 3:**
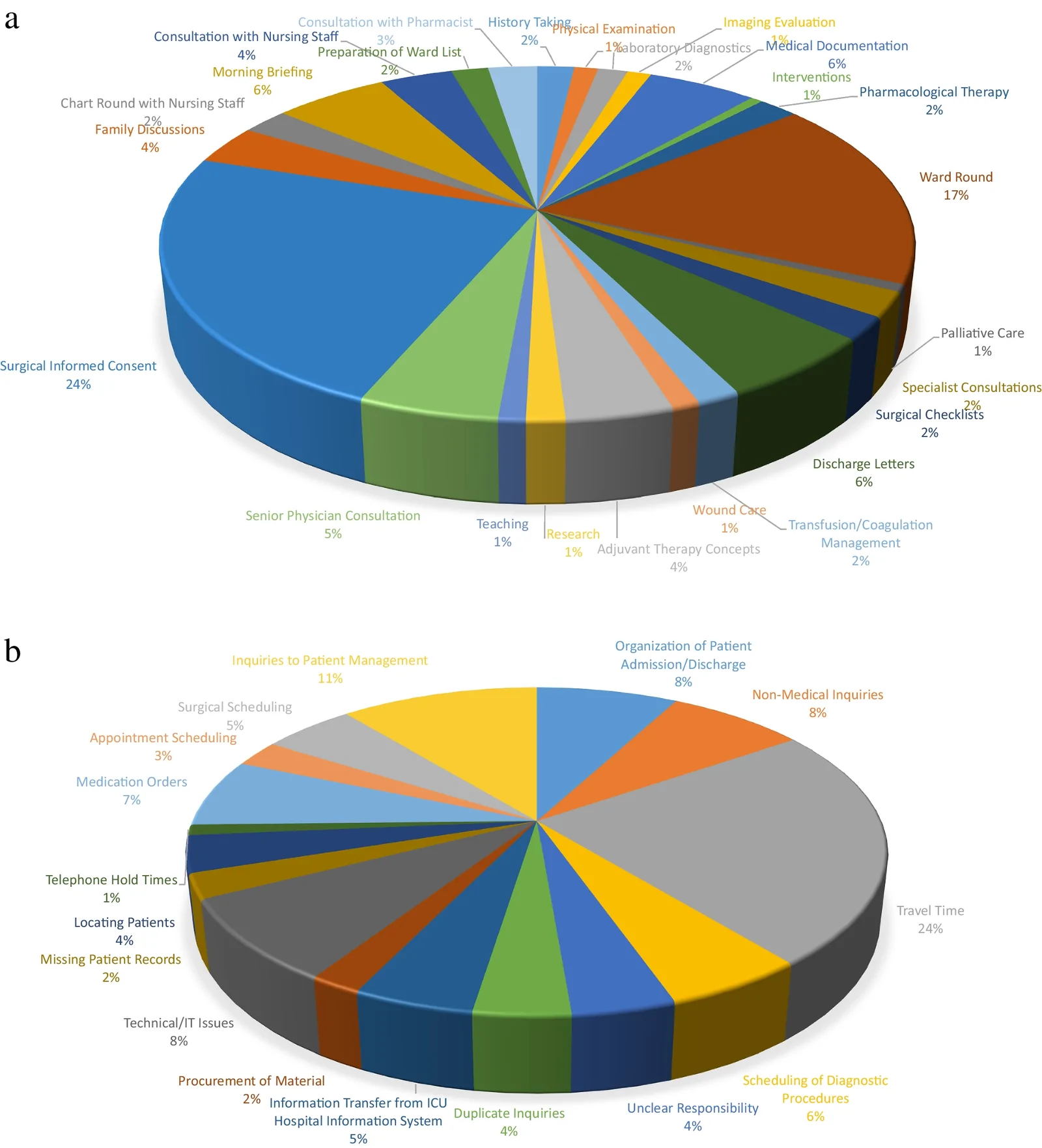
**a** illustrates the percentage distribution of direct and indirect physician tasks. **b** provides an overview of the percentage shares of non-physician tasks during the 24-h on-call duty shifts

Analysis revealed that 60.6% of total documented time was attributable to direct and indirect medical activities, while 24.7% was spent on non-medical tasks (including administrative duties, organizational processes, and logistical activities). Recovery time, meals, and brief interruptions such as restroom use accounted for 14.0% (Fig. 3a + *b*). The mean distance walked per 24-h on-call shift was 8643 m (± 3.457,5 m). The survey revealed that all residents reported feeling overburdened by non-physician tasks integrated into their clinical duties, resulting in physiological and psychological strain during shifts. A central finding concerned non-medical tasks, which consumed substantial time and thereby compromised patient care, with all residents indicating that they spent time on non-patient-care-related tasks "often" or "very often". Furthermore, all respondents reported having only one hour per week available for research and teaching (seminars, lectures). Despite these burdens, residents rated the quality of clinical training as high and reported adequate supervisory support. (*see supplementary material*).

(b) **General ward work**

**Fig. 4:**
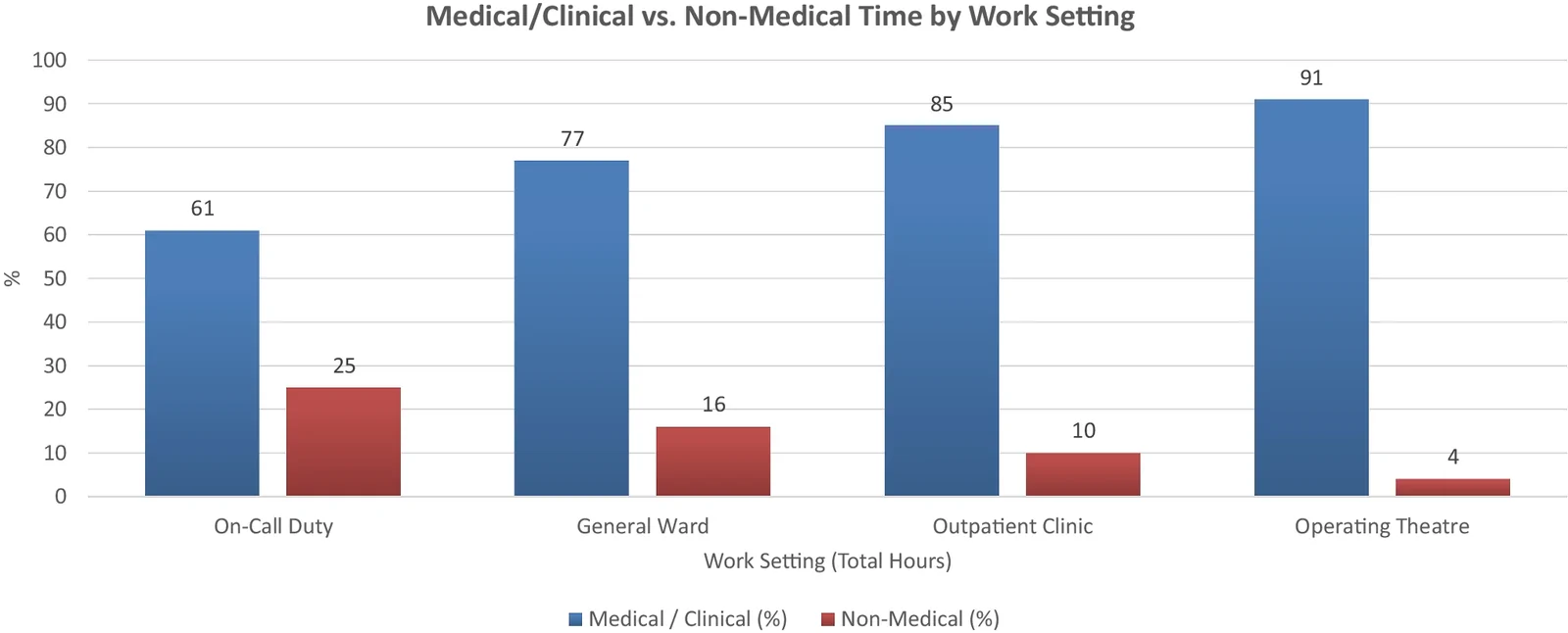
Overview of the proportions of physician and non-physician tasks during 24-hour shifts, on the general ward, in the outpatient clinic, and in the operating room

A total of 87 h and 35 min were documented on the general ward. Medical activities accounted for 76.6% of work time and non-medical activities for 15.6%. (Fig. 4) The largest proportion of medical work was dedicated to preoperative informed consent consultations (24%), ward rounds (17%), and discharge letter writing and clinical documentation (12%).

**Fig. 5:**
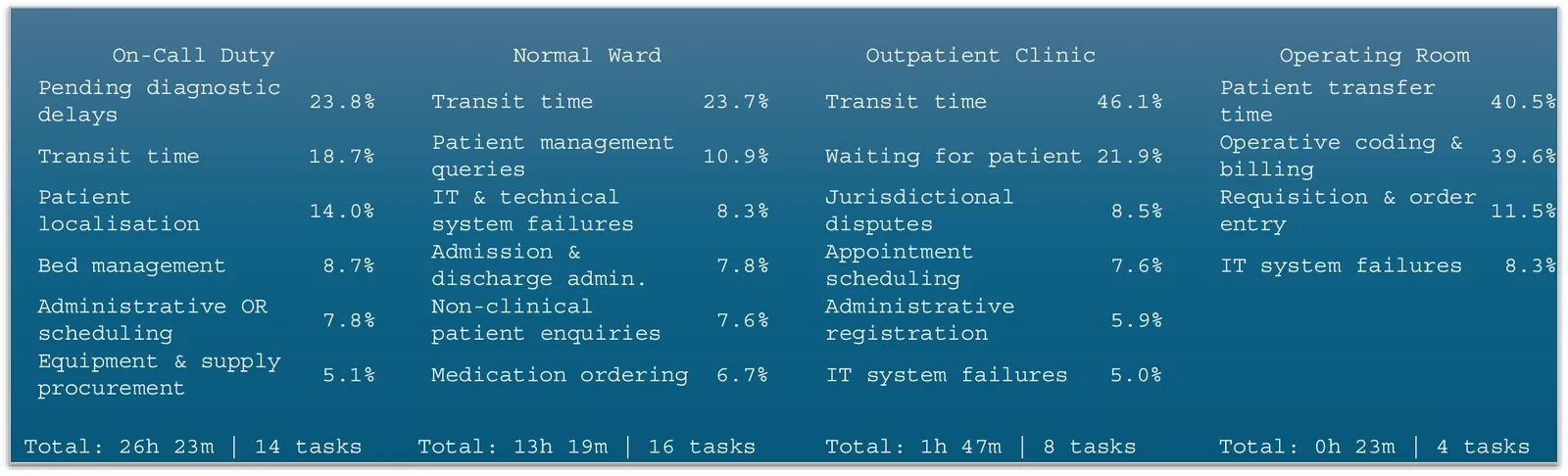
Distribution of the most frequent non-physician tasks during on-call duty, on the normal ward, in the outpatient clinic, and in the operating room

As observed in the on-call shifts, a noteworthy proportion of time (24%) was spent on transit between locations, in addition to time lost due to technical and IT system failures (8%), non-medical tasks including organizational aspects of patient admission and discharge (8%), organizational coordination with patient management (11%), and provision of non-medical information to patients or relatives (8% of non-medical activities). Within the context of ward work, the greatest share of knowledge transfer occurred through on-the-job training (60%), while mentoring accounted for at least 29% of learning time (Fig. 5).

(c) **Outpatient clinic**

Time recording covered a total of 35 h and 15 min, during which 84.8% of time was spent on medical activities and 9.9% on non-medical activities. (Fig. 4) The greatest proportion of time was devoted to direct patient contact, including history-taking and physical examination (44%). Other major activities comprised the evaluation of CT and MRI imaging (10%) and clinical documentation (17%). Time allocated to mentoring through senior physician consultation accounted for only 7%.

The highest time expenditure among non-medical activities and procedural inefficiencies was attributable to transit time (39%) and waiting for patients (16%) due to their unclear whereabouts within the hospital. Unresolved responsibilities (12%) and appointment scheduling (11%) constituted lesser but notable time expenditures (Fig. 5).

(d) **Operating theater**

The total documented time spent by resident physicians in the operating theater amounted to 32 h and 58 min, of which 90.5% was attributable to medical activities and 3.8% to non-medical activities. (Fig. 4) A substantial proportion of operating time (38%) involved active surgical participation by the resident under supervision, while 39% was spent on surgical assistance. The remaining time consisted of pre- and postoperative preparation and follow-up.

The most significant non-medical time expenditures in the operating theater arose from delayed patient transport (38%), turnover time between procedures (21%), and procedural coding (14%) (Fig. 5).

## Discussion

The physician staffing requirement derived from the InEK cost matrix provided only a partial reflection of the department's actual personnel costs [20]. The InEK calculation is primarily case- and DRG-based, and therefore captures predominantly direct medical activities [9]. However, a substantial portion of physician work consists of indirect activities that are not or only insufficiently reflected in DRG reimbursement, including postgraduate training and supervision, interdisciplinary coordination, committee and quality management work, research and teaching, the development and implementation of clinical guidelines, IT-related tasks, and extensive administrative communication [37, 38]. Additionally, the DRG system does not account for departmental subspecialization, which enables more complex procedures and requires specifically trained medical staff. In neurosurgery, this includes, for example, cerebrovascular surgery, skull base surgery, complex spinal surgery, functional neurosurgery, and pediatric neurosurgery.

Furthermore, InEK data are based on average values from participating hospitals, thereby tending to perpetuate existing staffing deficiencies or already "optimized" staffing structures rather than reflecting a normative staffing requirement. Developments in collective bargaining agreements, increasing on-call workload, growing complexity due to multimorbidity, and additional regulatory requirements (documentation and reporting obligations) are reflected only with a time lag or incompletely [19, 23, 36]. When the physician budget derived from the InEK matrix is subsequently converted into FTEs, the resulting figure represents the number of theoretically fundable rather than operationally required physician posts. As a consequence, the InEK calculation systematically excludes key workload drivers, resulting in chronic understaffing of physician personnel, particularly in departments with a pronounced commitment to postgraduate training, research, or academic medicine [6, 39].

The deductive-institutional cooperative approach, developed in close collaboration between personnel controlling and the clinical department, yielded a higher number of required FTEs, as numerous clearly defined indirect medical activities were transparently accounted for. The model developed by the German Medical Association (Bundesärztekammer) is comparable to the WISN model (Workload Indicators of Staffing Need), which was developed by the WHO as early as the 1990 s to enable evidence-based assessment of personnel requirements in healthcare [5, 24]. Pending the results of the ongoing ÄPS-BÄK assessment procedure, the available evidence suggests that this modeling approach more accurately reflects the demands of physician work. By integrating department-specific contextual data, care pathways, interaction density, and structural characteristics, this approach is better suited to capturing complex physician activity profiles. At university hospitals in particular, characterized by high interdisciplinarity, substantial research obligations, and emergency care provision, this model type appears considerably closer to the realities of everyday clinical practice than the InEK calculation. Nevertheless, neither model accounts for research rotations at university hospitals, which represent an essential component of academic medical training and the ongoing development of the specialty. This factor should likewise be incorporated into staffing calculations for academic institutions.

Furthermore, it must be emphasized that neither the InEK model nor the deductive-institutional approach adequately captures the major workload-contributing factors encountered in clinical practice. Numerous confounding factors represent substantial drivers of time expenditure and occupational burden in the daily work of physicians. Although these activities are not intrinsically medical in nature, they are routinely performed by physicians in the absence of systemic alternatives [4, 10, 12, 25]. Without systematic consideration of these factors, any assessment of actual workload will inevitably remain distorted. In the present study, we demonstrated that approximately 30% of activities performed during on-call shifts were non-physician in nature. This observation is consistent with the findings of a multicenter observational study conducted in the United Kingdom (UK), which similarly demonstrated that 73% of trainees' tasks did not involve direct patient contact [4]. There is therefore a clear need to expand future staffing assessment instruments to encompass such confounding factors, through the integration of a structured deductive approach, a realistic capture of both core and peripheral physician activities, and the site-specific systematic quantification of non-medical tasks currently performed by physicians. As long as these structural conditions remain unaddressed, the explicit consideration of such confounding factors is indispensable to ensuring a staffing environment that is both needs-based and conducive to physician well-being [16, 27].

Irrespective of whether and how the workload generated by confounding factors and operational inefficiencies is reimbursed, our analysis demonstrates that these burdens can be precisely identified and quantified through systematic time-and-activity documentation [28, 33]. This evidence base can be used to target those processes that are amenable to optimization with comparatively modest effort, yet whose improvement would yield immediately perceptible gains in efficiency, job satisfaction, and reduction of error propensity. Practical examples include centralized, digitally managed bed allocation systems, largely automated surgical scheduling with standardized interfaces to anesthesiology, radiology, and nursing, and the deployment of electronic real-time patient tracking systems that enable the precise localization of patients throughout the hospital, thereby minimizing time-consuming inquiries and unnecessary transit [31]. Modern hospitals are increasingly equipped with digital patient tracking systems that allow real-time localization and process monitoring [34]. International studies have demonstrated that patient tracking systems not only enhance operational efficiency but also improve patient safety by reducing waiting times, minimizing the risk of patient misidentification, and preventing process interruptions [11, 15, 17]. However, the benefit of such systems depends strongly on local infrastructure, architectural conditions, and integration with existing IT systems, such that each institution must evaluate their implementation individually. We have identified the following four strategies to reduce the time burden associated with non-physician tasks: (1) delegation (expanding the transfer of administrative tasks to non-physician staff), (2) digitalization (real-time reporting of findings, automated discharge management), (3) enhanced transparency (patient-tracking systems, shared multidisciplinary task lists within the hospital information system), and (4) increasing centralization (bed management, discharge management, and operating room scheduling). Colleagues from Toronto [14] demonstrated, in a surgical team at a tertiary care center, that delegating physician tasks to Physician Assistants led to a significant improvement in quality of care and a reduction in resident workload. At our institution, as in Canada and the United States, Physician Assistants are employed to support ward-based work. This may also help explain why non-clinical activity on the ward is comparatively lower than during on-call duty. In many German hospitals, however, Physician Assistants are not yet routinely employed.

Only by creating a clinical environment with fewer confounding factors and operational disruptions can temporal capacity be generated to allow for the active and intentional delivery of postgraduate medical education. In this context, mentoring, defined as the structured transmission of knowledge and clinical reasoning by experienced colleagues, deserves particular emphasis, as it has been shown to contribute demonstrably to the quality of medical training. Learning on the job and peer learning also represent important and evidence-supported modalities of clinical learning that meaningfully shape professional development. Nonetheless, excessive workload during on-call duties can render physicians vulnerable to psychological and mental health burdens. In recent years, the increasing integration of artificial intelligence, virtual reality, and advanced information technology has contributed to improvements in the quality of medical education. Established learning modalities such as cadaveric courses and simulation-based training likewise continue to make a substantial contribution to knowledge acquisition and safe clinical performance. The high quality of postgraduate training must therefore not be undermined by an insufficient quantity of dedicated training opportunities.

### Limitations

The findings presented here are not directly generalizable and should be regarded primarily as an illustration of a methodological approach. This is a single-center study and therefore cannot reflect the workload or working conditions of resident physicians in other departments or countries. The working conditions underlying our model reflect the regulatory framework of the EU Working Time Directive and national regulations. It should be adapted accordingly when applied to healthcare systems governed by different working-time regulations. Furthermore, workload is substantially dependent on departmental staffing levels, case mix, and local organizational structures, all of which are subject to fluctuation. Other institutions are encouraged to conduct their own data-driven analyses of site-specific operational inefficiencies to identify local optimization potential and derive tailored improvement measures.

Nevertheless, while the absolute staffing requirements reported here are specific to the investigated institution and the German healthcare system, the underlying challenges are likely not unique to this setting. Neurosurgical departments worldwide must balance direct patient care with administrative responsibilities, organizational tasks, on-call commitments, and other non-clinical duties. Therefore, the methodological framework presented in this study may also be useful for identifying inefficiencies and optimizing physician workforce allocation in other healthcare systems.

## Conclusions

A combination of overarching staffing assessment instruments and site-specific process analysis is essential to achieving a physician staffing configuration that is realistic, health-promoting, and economically sustainable in the long term, while also creating the temporal capacity necessary for the active delivery of postgraduate training in everyday clinical practice. Although staffing requirements must ultimately be determined locally, our findings suggest that a substantial proportion of physician workload arises from indirect clinical, organizational, and administrative activities that are insufficiently captured by conventional staffing models. As these challenges are likely shared by neurosurgical departments across different healthcare systems, systematic identification and reduction of non-clinical workload may represent an important opportunity to improve workforce utilization and maintain high-quality neurosurgical care internationally.

## Supplementary Information

Below is the link to the electronic supplementary material.

Supplementary file1 (PDF 130 KB)

## Data Availability

No datasets were generated or analysed during the current study.
