# Supplementary material for: Beyond clinical care: direct, indirect, and non-physician activities and their implications for workforce planning and postgraduate medical education in a university neurosurgical department
Source: Acta Neurochir (Wien). 2026 Nov 13;168(1):262. doi: 10.1007/s00701-026-07084-y (PMC13769772; doi:10.1007/s00701-026-07084-y)
Supplement: Supplementary file 1 [file 701_2026_7084_MOESM1_ESM.pdf]

	all residents	
Part 1: General Information		
1. Age		
under 25	0	0.0%
25-30	3	33.3%
31-35	5	55.6%
36-40	1	11.1%
over 40	0	0.0%
2. Gender		
female	5	55.6%
male	4	44.4%
3. Residency since		
less than 1 year	0	0.0%
1-2 years	2	22.2%
3-4 years	4	44.4%
5 years or more	3	33.3%
Part 2: Evaluation of training quality		
4.How would you rate the quality of training in your neurosurgical shift?		
very good	0	0.0%
good	4	44.4%
satisfactory	4	44.4%
sufficient	1	11.1%
unsatisfacory	0	0.0%
5. How often is it possible to participate in surgeries actively?		
very often	2	22.2%
often	6	66.7%
sometimes	1	11.1%
rarely	0	0.0%
never	0	0.0%
6. How would you rate the support from your superior during your training?		
Very good	2	22.2%
Good	3	33.3%
Satisfactory	3	33.3%
Sufficent	1	11.1%
Unsatisfactory	0	0.0%
7. Dou you feel adequately prepared to perform surgeries on your own?		
Yes. completely	1	11.1%
Yes. mostly	3	33.3%
Partly	3	33.3%
No. not really	2	22.2%
No. not at all	0	0.0%
Part 3: Psychological and physical stress during shifts		
8. How would you rate the general psychological and physical stress during your shifts?		
Very high	3	33.3%
High	3	33.3%
Medium	2	22.2%
Low	1	11.1%
Very low	0	0.0%
9. How often do you feel physically exhausted during your shift?		
Very often	3	33.3%
Often	4	44.4%
Sometimes	1	11.1%
Rarely	1	11.1%
Never	0	0.0%
10. Do you have the feeling that your mental health is affectet by your neurosurcical shift?		
Yes, strongly	1	11.1%
Yes, slightly	7	77.8%
Neutral	0	0.0%
No, not really	0	0.0%
No, not at all	1	11.1%
Part 4: Non-medical tasks		
11. How much time (in hours per week) do you spend on non-medical tasks (documentation. calls. organizational tasks. ...)?		
0-2 hours	0	0.0%
3-5 hours	2	22.2%
6-10 hours	4	44.4%
11-15 hours	0	0.0%
over 15 hours	3	33.3%
12. Which non-medical tasks take most of your time?		
Dokumentation	8	57.1%
Calls	4	28.6%
Organisation of transports	1	7.1%
Technical defects	1	7.1%
Part 5: taking on tasks		

	all residents	
Part 6: Effects on training and workload		
16. How does overtaking these tasks affect your training?		
Very positive	0	0.0%
Positive	0	0.0%
Neutral	1	11.1%
Negative	7	77.8%
Very negative	1	11.1%
17. Do you feel overburdened with overtaking these tasks?		
Yes, strongly	1	11.1%
Yes, slightly	5	55.6%
Neutral	3	33.3%
No, not really	0	0.0%
No, not at all	0	0.0%
18. How does overtaking tasks from other occupational groups influence your time for clinical tasks?		
Very negative	1	11.1%
Negative	7	77.8%
Neutral	1	11.1%
Positive	0	0.0%
Very positive	0	0.0%
Part 7: Suggestions for improvement		
19. What measures would you think would help to reduce taking over tasks from other occupational groups?		
Improvement of interdisciplinary communication	5	21.7%
Clear definition of responsibility	8	34.8%
Increase the staff in other occupational groups	6	26.1%
Trainings for residents regarding better delegation of tasks	2	8.7%
More employees in own occupational group	1	4.3%
Higher quality standards for the training of other occupational groups (for example nursing staff)	1	4.3%
Part 7: Identification of time wasters		
20. How often do you experience problems regarding IT?		
Very often	4	44.4%
Often	5	55.6%
Sometimes	0	0.0%
Rarely	0	0.0%
Never	0	0.0%
21. How much time (in hours per week) do you spend with transportation (patient transportation, transportation of materials, ...)?		
0-2 hours	7	77.8%
3-5 hours	2	22.2%
6-10 hours	0	0.0%
11-15 hours	0	0.0%
more than 15 hours	0	0.0%
22. How often do you have difficulties regarding bed management (no beds available, long waiting time, ...)?		
Very often	3	33.3%
Often	6	66.7%
Sometimes	0	0.0%
Rarely	0	0.0%
Never	0	0.0%
23. How often do you spend time with calls which don't have a direct link to patient care?		
Very often	4	44.4%
Often	5	55.6%
Sometimes	0	0.0%
Rarely	0	0.0%
Never	0	0.0%
24. Do you often have missing access to patient files because of data protection regulations?		
Yes, very often	3	33.3%
Yes, often	3	33.3%
Sometimes	1	11.1%
Rarely	2	22.2%
Never	0	0.0%
25. How often do you experience difficulties because of parted facilities (East-West, off-site facilities, ...)?		
Very often	4	44.4%
Often	5	55.6%
Sometimes	0	0.0%
Rarely	0	0.0%
Never	0	0.0%
Part 9: Impact on research. teaching and self-study		
26. How much time remains for research per week?		
0-1 hour	9	100.0%
2-3 hours	0	0.0%
4-5 hours	0	0.0%
6-10 hours	0	0.0%
more than 10 hours	0	0.0%

	all residents			all residents	
13. How often do you take over tasks. which would normally be done by another occupational group (nursing staff, therapists, administration, ...)?			27. How much time remains for teaching (seminars, lectures, ...) per week?		
Very often	2	22.2%	0-1 hour	9	100.0%
Often	6	66.7%	2-3 hours	0	0.0%
Sometimes	1	11.1%	4-5 hours	0	0.0%
Rarely	0	0.0%	6-10 hours	0	0.0%
Never	0	0.0%	More than 10 hours	0	0.0%
14. Which tasks do you take over most frequently?			28. How much time remains for self-study per week?		
Documentation of patient data	2	10.5%	0-1 hour	8	88.9%
Organisation of transports	5	26.3%	2 hours	1	11.1%
Perform administrative tasks	7	36.8%			
Communication with relatives	5	26.3%			
Support in patient care (for example during mobilisation)	0	0.0%			
Telephone book instead of reception desk	0	0.0%			
15. How much time (in hours per week) does it consume to take over these tasks?					
0-2 hours	3	33.3%			
3-5 hours	4	44.4%			
6-10 hours	1	11.1%			
11-15 hours	1	11.1%			
more than 15 hours	0	0.0%			
